# Usability and Satisfaction Outcomes from a Pilot Open Trial Examining Remote Patient Monitoring to Treat Pediatric Obesity during the COVID-19 Pandemic

**DOI:** 10.3390/ijerph20032373

**Published:** 2023-01-29

**Authors:** Crystal S. Lim, Cameronne A. Dodd, Laura E. Rutledge, Shanda W. Sandridge, Krista B. King, Darryl J. Jefferson, Tanya Tucker

**Affiliations:** 1 Department of Health Psychology, University of Missouri, Columbia, MO 65203, USA; 2 Cancer Center and Research Institute, University of Mississippi Medical Center, Jackson, MS 39216, USA; 3 Department of Psychiatry and Human Behavior, University of Mississippi Medical Center, Jackson, MS 39216, USA; 4 Department of Pediatrics, University of Mississippi Medical Center, Jackson, MS 39216, USA; 5 Center for Telehealth, University of Mississippi Medical Center, Jackson, MS 39216, USA

**Keywords:** telemedicine, pediatric obesity, remote patient monitoring, weight management

## Abstract

Background: Pediatric obesity is common and a significant burden. Supplementing pediatric obesity treatment with technology is needed. This manuscript examines the usability and satisfaction, as well as explores initial effectiveness, of a remote patient monitoring system (RPMS) designed for youth presenting for pediatric weight management treatment. Methods: 47 youth, 10 to 17 years old, with obesity and a caregiver participated. For three months, families received treatment via the RPMS. Usability and satisfaction outcomes were examined. Exploratory analyses were conducted to examine initial effectiveness from baseline and post-treatment (month 3) assessments. Results: More than 80% of patients used the RPMS, and overall, patients completed 27 out of 90 daily sessions (30%). Youth and caregivers reported high satisfaction. Non-parametric tests revealed no significant improvements for youth or caregiver weight status after the RPMS treatment. Significant improvements in other outcomes examined were limited. Conclusions: Families were satisfied with the RPMS, but use of the system was limited. Initial effectiveness was not able to be determined due to the amount of missing data, which was impacted by the COVID-19 pandemic. Modifications of the RPMS and future evaluation of usability and effectiveness are warranted to determine utility in supplementing pediatric obesity clinical treatment.

## 1. Introduction

In the United States (US), pediatric obesity is an important health concern due to its prevalence and negative impacts. About one in five youth 6–19 years of age in the US are obese, and more than 5% have severe obesity [1]. Increased risk of serious chronic health conditions, like type 2 diabetes, is associated with obesity in childhood [2]. Pediatric obesity is a burden on health care systems [3]; for example, it is estimated to account for over USD 14 billion a year in health care costs [4]. There is a need for treatments focused on pediatric obesity in order to reduce the development of comorbidities and lower health care costs [5].

Outpatient pediatric obesity clinics have demonstrated improvements in weight status for about 50% of youth receiving treatment [6,7]. However, providing consistent and efficient care is more difficult when health care providers treat children and families from underserved and under-resourced backgrounds [8,9]. Mississippi is a mostly rural state and has the largest population of non-Hispanic Black/African Americans (>35%), and more than one in three youth live in poverty [10]. Patients living in Mississippi also experience numerous health disparities, including pediatric obesity prevalence rates that are the highest in the country [11]. Thus, there is a significant need to develop and evaluate technology-based interventions that have the potential to reduce health disparities and improve weight status in youth living in Mississippi [5].

Use of telemedicine in health care delivery has increased due to rising cell phone, tablet, and computer use, as well as more widespread availability of internet and Wi-Fi access [12]. Remote patient monitoring systems (RPMSs) have been developed and implemented by hospital systems and insurance companies to provide treatment and support for patients with chronic medical conditions [12]. In general, RPMSs medically monitor health outcomes via electronic devices, can be a source of patient education, and have capabilities to alert health care teams and patients to potential medical problems that need immediate attention [12]. Studies evaluating RPMSs have revealed improved health in adults with various chronic medical conditions [13,14,15], including obesity [16]. However, the application of RPMSs for use in pediatric populations has been limited. To date, we are aware of four published studies that examined RPMSs for pediatric health conditions [17,18,19,20]. In general, feasibility, effectiveness, and reduced health care costs were found; however, we are not aware of RPMSs being previously developed and implemented for pediatric obesity treatment. RPMSs have the potential to provide children and their families with additional education about healthy lifestyle behaviors and behavioral change strategies applicable to pediatric obesity in between clinic visits, as well as provide additional support from the health care team if needed.

The purpose of this paper is to report initial outcomes of a pilot open trial evaluating an RPMS that was developed to supplement care for youth attending an outpatient specialty pediatric weight management clinic. Usability and satisfaction were examined, and the initial effectiveness of the RPMS was explored. Our hypothesis was that high feasibility regarding use of the RPMS would be found and satisfaction rates would be over 75%. In addition, we expected that after using the RPMS for three months, participating youth would exhibit significant improvements in weight status and health behaviors, and caregivers would report improved child quality of life and home food environment, as well as exhibit healthier weight status compared to pre-treatment.

## 2. Materials and Methods

The study is registered at ClinicalTrials.gov (Identifier: NCT04029597) and was approved by the University of Mississippi Medical Center (UMMC) Institutional Review Board (IRB) (Study #2017-0083). Detailed design and methods have been previously reported [5]. In summary, we utilized an open trial pilot design. Recruitment began before the COVID-19 pandemic. However, additional patients were recruited, and post-treatment and follow-up assessments were completed during the pandemic using IRB-approved modified remote strategies, such as via mail and phone.

### 2.1. Participants

Participants included 47 families with a child 10 to 17 years of age attending a regularly scheduled outpatient multidisciplinary pediatric obesity clinic visit. Inclusion criteria included youth weight status in the obese range (i.e., body mass index [BMI] equal to or above the 95th percentile based on age and gender) and caregiver and youth fluency in English. Exclusion criteria consisted of: (1) history of cognitive impairment (developmental delay or intellectual disability) for youth or caregiver and/or (2) youth medical condition that may prohibit wearing an actigraph device reported by caregiver. Eligible families were informed about the study during the outpatient clinic appointment. Clinic providers gave families information about the study during the visit, and if families were interested in more information, a trained research assistant discussed the project in more detail and obtained youth assent and caregiver permission and consent. Youth and caregivers then completed baseline questionnaires and assessments during the clinic visit. Due to poor retention of families at the 3-month follow-up assessment, we only examined RPMS use data, satisfaction, and efficacy results from the pre-treatment and post-treatment assessments. In addition, due to the fact that many of the post-assessments were conducted remotely due to the COVID-19 pandemic, health outcomes collected during routine in-person clinic visits (e.g., glucose, hemoglobin A1c) could not be obtained. Thus, those data are not examined in the current manuscript. 

### 2.2. Remote Patient Monitoring

The RPMS was developed in consultation with the UMMC Center of Telehealth and based on a group-based behavioral family weight management intervention previously implemented in the pediatric weight management clinic. Details of the RPMS are reported elsewhere [5]. In summary, the RPMS developed and examined in this study consisted of a tablet device, scale to measure weight, and pedometer to assess physical activity. Educational material was presented daily via brief presentations and video clips focused on healthy eating, physical activity, and other health and behavioral topics. Examples of topics included: importance of exercise, how to monitor eating, reviewing food groups, portion sizes and reading nutrition labels, how to set goals, identifying eating cues, and the importance of sleep. Patients interacted with the UMMC Center for Telehealth nurse care coordinators and research and clinical staff on an as-needed basis during the three-month RPMS treatment period. 

## 3. Measures

Weight Status. Youth weight status (e.g., BMI, BMI z-scores, and BMI percentiles using age- and gender-specific norms) and caregiver weight status (i.e., BMI) were calculated using height and weight measurements obtained from standard clinical procedures implemented by trained clinical staff or research personnel. 

Health Outcomes. Trained nursing professionals in the pediatric obesity clinic assessed youth blood pressure and heart rate. We planned to examine results from routine blood work to assess changes in glucose and hemoglobin A1c, but these data were only available for a small portion of the participating youth for both the pre-treatment and post-treatment assessments (Glucose *n* = 4 and hemoglobin A1c *n* = 7), so analyses were not conducted on these data for the current manuscript. 

Demographic Information. Caregivers completed a demographic questionnaire at the pre-treatment assessment to gather general information about the youth and caregiver (e.g., age, sex, race/ethnicity). 

Dietary Intake. To assess dietary intake, youth completed a web-based eating recall using the Automated Self-administered 24-h Recall (ASA24). The ASA24-2018 was developed for participants 10 years and older and guides youth through the 24 h of the previous day by asking what foods were eaten and the estimated portion sizes consumed for each meal and snack. Responses were scored using the USDA’s Food and Nutrient Database for Dietary Studies. The primary dietary intake variable examined was total kilocalories (kcals) per day. 

Physical Activity. To examine physical activity, participating youth were given an individually programmed ActiGraph GT3X+ Activity Monitor [21] to wear for one week after each assessment. Families returned the actigraph to study staff via a stamped envelope after the device was worn for seven days. For the purposes of this study, average kcals from physical activity was the variable examined in primary analyses.

Self-efficacy. Youth completed the Child Dietary Self-Efficacy Scale (CDSES) [22] and the Self-Efficacy for Physical Activity Scale (SEPAS) [23] to examine healthy eating self-efficacy and ways they manage physical activity barriers, respectively. The CDSES includes 15 items, and the SEPAS contains five items. Both measures have been used previously in pediatric research and have acceptable reliability and validity [22,23]. In this sample, Cronbach’s alpha for the CDSES was 0.84 and was 0.71 for the SEPAS.

Quality of Life. The Pediatric Quality of Life Inventory (PedsQL) [24] child report and parent report versions were utilized to assess youth quality of life. The PedsQL consists of 23 items and has demonstrated good reliability and validity in a variety of pediatric populations [24]. The Total PedsQL score from the child and parent report version of the questionnaire were the primary quality of life variables examined in the current study. Reliability for both versions of the questionnaire was good in the current sample (child report PedsQL total Cronbach’s alpha = 0.87; parent report PedsQL total Cronbach’s alpha = 0.84). 

Home Food Environment. Caregivers completed the Home Food Inventory (HFI) [25] to examine healthy and unhealthy foods available in the family’s home. The measure has previously demonstrated adequate reliability and validity in health promotion research. For the current study, 71 yes or no questions comprising the obeseogenic home food availability score was used. Possible scores range from 0–71, with higher scores indicating increased access to unhealthy foods. In the current sample, the scale demonstrated good reliability (Cronbach’s alpha = 0.89).

RPMS Use. Information regarding the number of partially and fully completed RPMS sessions completed by each patient was extracted from the RPMS system. RPMS sessions were scheduled every day for 3 months; thus, the number of planned RPMS sessions was 90. 

Treatment Satisfaction. The Treatment Satisfaction questionnaire was completed by youth and caregivers at the post-treatment assessment. The questionnaire was developed specifically to examine satisfaction with the RPMS for this study and was based on previous research [26]. The youth version consists of 10 statements, and the caregiver version consists of 12 statements about the RPMS. Participants rated how much they disagreed or agreed with each statement on a 4-point Likert scale, with higher scores indicating more satisfaction with the RPMS. Both the youth and caregiver versions of the treatment satisfaction questionnaire demonstrated good reliability (Youth Treatment Satisfaction Cronbach’s alpha = 0.82; Caregiver Treatment Satisfaction Cronbach’s alpha = 0.85). 

### 3.1. Compensation

Families received compensation for completing each assessment during the 6-month study period (e.g., $20 pre-assessment, $30 post-assessment, and $40 follow-up assessment).

### 3.2. Data Analysis Plan

To examine RPMS usability and satisfaction, descriptive statistics (*M, SD*, *N,* %) regarding use of the RPMS and satisfaction scores from the youth and caregiver treatment satisfaction questionnaire were implemented. To explore the initial effectiveness of the RPMS, we planned to conduct paired samples *t*-tests to compare pre-treatment to post-treatment differences in the primary outcomes of interest. However, most of the effectiveness outcomes of interest were not normally distributed, so non-parametric tests, specifically Wilcoxon signed ranks tests, were used. Missing values were left missing in analyses. SPSS was used to conduct analyses, and statistical significance was set at *p* < 0.05. 

## 4. Results

### 4.1. Preliminary Analyses

Table 1 displays youth and caregiver demographic characteristics of the sample recruited to participate in the study (*N* = 47). Youth ranged in age from 10 to 17 years, were primarily female (57.4%) and identified as non-Hispanic Black (70.2%). In terms of youth obesity severity, most had Class 3 Obesity (*n* = 23, 48.9%). Caregivers (*N* = 45) ranged in age from 30 to 70 years, and most (86.7%) were the participating child’s mother. Caregivers were primarily single/never married (37.8%) or married (35.6%), and 66.7% (*n* = 30) reported a family income below $50,000. Weight status was available for 35 out of the 45 participating caregivers. In terms of caregiver weight status, 85.7% (*n* = 30) were obese, with 12 (34.3%) having Class 3 Obesity. Out of the 47 youth who were enrolled and completed the pre-treatment assessment, 38 (80.9%) used the RPMS, and 30 (63.8%) completed the post-assessment. Reasons for not initiating the RPMS included: one child was diagnosed with type 2 diabetes and initiated use of the type 2 diabetes RPMS, *n* = 3 discontinued use of the RPMS before the 3-month treatment period ended, and *n* = 5 did not receive or initiate use of the RPMS. Reasons for not completing the post-treatment assessment included: *n* = 8 were unable to contact/did not return questionnaires after repeated attempts. 

### 4.2. Engagement and Satisfaction

Engagement with RPMS. The summary of participant engagement with the RPMS is in Table 2. Data were available for 38 patients who used the RPMS. On average, youth participated in 27 RPMS sessions during the 3-month period out of 90 possible sessions (30.3%). The number of RPMS sessions completed ranged from 0 to 88, and the median, or most common number, of completed sessions was 24.50. Thus, engagement was below the a priori identified target of 75%.

Satisfaction with RPMS. The descriptive results from youth and caregiver reports on the RPMS treatment satisfaction questionnaires are presented in Table 2. Overall, total treatment satisfaction rates were 82.0% in youth and 88.0% in caregivers, which is higher than our a priori identified acceptable levels of satisfaction (e.g., 75%). Table 2 also details the descriptive statistics for each question included on the treatment satisfaction questionnaires. Youth indicated that they were most satisfied with how easy it was to answer questions in the RPMS, and they were least satisfied with how time-consuming the RPMS was. For caregivers, they identified being the most satisfied being comfortable answering questions asked by the RPMS and the system’s ease of use. Caregivers were least satisfied with the RPMS helping them improve their child’s behavior. 

### 4.3. Exploratory Analyses 

Initial Effectiveness. Exploratory results examining the initial effectiveness of the RPMS are presented in Table 3. Results revealed no significant pre-treatment to post-treatment improvements in youth weight status (e.g., BMI z-score, BMI percentile, or BMI), which was the primary outcome of interest. Analyses revealed significant improvements in total quality of life based on youth report (Z = −2.114, *p* = 0.035). However, results also revealed significant worsening in engagement in physical activity and obesogenic home food availability. There were also no significant differences found for heart rate, blood pressure, dietary intake, healthy eating self-efficacy, physical activity self-efficacy, caregiver weight status, or caregiver report of youth total quality of life. If corrections for multiple comparisons are taken into account, none of the results would have reached significance (e.g., using Bonferroni correction, significance would be set at *p* < 0.004). 

## 5. Discussion

In this pilot study, an open trial design was used to examine the initial usability and satisfaction of an RPMS designed for youth receiving pediatric obesity treatment in an outpatient specialty medical clinic. The initial effectiveness of the RPMS was also explored. There is a lack of accessible treatments that address severe pediatric obesity for youth from rural and underserved areas [5]. Bariatric surgery is effective [27,28], but in the clinic where the study was conducted, it was not available when the RPMS was developed. Technology-based treatments, such as RPMSs, appear promising in treating adults and youth with various chronic medical conditions and may be one avenue to provide more intensive and effective treatments to address severe pediatric obesity.

Results regarding the usability and satisfaction of the RPMS were mixed. Youth and caregivers who completed the post-treatment assessment reported high satisfaction with the RPMS. However, only 80% of families who initially consented to participate in the study used the RPMS, and only 63% of families completed the post-treatment assessment. In addition, for those who used the RPMS, use was less than we hoped, given that on average youth completed about 30% of the daily sessions. The potential role of the COVID-19 pandemic on the use of the RPMS cannot be overlooked. The pandemic disrupted recruitment and retention of families in this study, which has been documented in the literature [29]. Specifically, recruitment started prior to the pandemic, and then, due to institutional constraints, study pre-treatment and post-treatment assessments were converted to completion through the mail or over the phone. Assessments were initially intended to be scheduled during in-person visits in the pediatric obesity clinic, but at the start of the COVID-19 pandemic, clinic visits were converted to telehealth appointments, which led to limited face-to-face interaction with families and missing data such as height and weight measurements and other clinical measures (e.g., blood pressure, heart rate, routine blood work). Given the context families were operating in during the pandemic, such as virtual school, the pandemic may also have influenced interest in engaging with another technological device. In addition, many of the families enrolled in the study were difficult to reach via phone (e.g., disconnected numbers, changed numbers) and email, which may have been exacerbated by factors related to the pandemic, such as loss of family income, school and work disruptions, and competing demands regarding health and safety [30,31]. 

In terms of initial effectiveness, conclusions that we can draw are limited due to the amount of missing data. However, in general, we found no improvements in youth or caregiver weight status after receiving the RPMS, which were the primary outcomes of interest. Given limited engagement with the RPMS, this may explain the lack of change in youth weight status. In addition, the short time of the intensive treatment (daily for 3 months) may not be a long enough period to see improvements in weight status for youth with severe obesity. Furthermore, the RPMS was not intended to directly address caregiver healthy lifestyle behaviors and weight, so improvements in caregiver BMI may not be expected. However, the finding that after receiving the RPMS there were significant improvements in youth-reported quality of life was promising. This finding is consistent with other pediatric weight management interventions where participating youth experience improvements in psychosocial outcomes [32]. However, these findings should also be considered in the context of results indicating worse lifestyle behaviors and health after the RPMS. Specifically, worse physical activity and home food environment after the RPMS are concerning. This finding may also be explained by the COVID-19 pandemic, consistent with what others have found [33]. The limited findings regarding the effectiveness of the RPMS for pediatric obesity warrant additional investigation.

Generally, the RPMS is considered an innovative application of integrating technology to deliver health care. For example, RPMSs are typically connected to electronic medical records and for patients and caregivers can provide quicker and more convenient access to health care education and health care providers. The RPMS examined in this study provided daily education specific to changing eating and physical activity behaviors over a three-month period for youth with severe obesity who need intensive approaches that are not typically available in pediatric outpatient clinics. However, it is possible the daily information and materials, even though presented in a brief format, were too intensive for the patients who participated. In addition, ways to further improve the RPMS based on treatment satisfaction scores and qualitative feedback were also identified and will be considered in future iterations and evaluation of the RPMS. The U.S. Preventative Task Force [34] recommends 26 h of treatment a year for pediatric obesity; however, pediatric obesity medical clinics are typically not able to provide that amount of treatment due to multiple institutional factors related to competing time, space, and other economic issues. Behavioral family interventions (BFIs) have been developed that provide group-based treatment in various clinical and community-based settings and are well-established, effective treatments for school-age children [35]. The RPMS developed for this study was based on previous group-based BFIs provided in the pediatric obesity clinic [36,37]. However, the RPMS was not as interactive with staff and providers as initially designed, which was in part due to the pandemic, as well as other logistical factors such as availability of telehealth nurses to interact with patients. Recent research has developed and implemented BFIs virtually for families living in rural areas [38,39]. One potential refinement of the RPMS that may increase engagement and effectiveness could be combining education materials and virtual groups, which may increase interactions between patients and the health care team. Given that RPMSs typically include a tablet with video capabilities, this could be a feasible modification to evaluate in future research. This combined RPMS may be a feasible, likeable, and effective telehealth intervention, as well as be a realistic avenue for clinics treating pediatric obesity to provide the evidence-based recommended amount of treatment.

Potential limitations of the current study and directions for future research are important to consider. First, the sample size was relatively small for this open trial pilot study. However, compared to previously published pediatric RPMS studies, our sample size was larger [20]. Furthermore, the large amount of missing data in this study limits conclusions that can be made regarding the usability, satisfaction, and effectiveness of the RPMS. There are also limitations regarding the assessments of lifestyle behaviors utilized in the current study, such as the 24-h dietary recalls completed via a web interface. Future research on a modified RPMS would be warranted with a larger sample size, a randomized controlled trial design, and inclusion of different lifestyle behavior assessment techniques. It would also be beneficial for future research to consider implementing intent-to-treat analyses and other multiple-imputation techniques to handle missing data in future studies examining RPMSs in this population. Second, the three-month length of RPMS treatment included in this study is a possible limitation. The designed RPMS was intensive (i.e., every day); however, the treatment length may not provide sufficient time to result in weight status or behavior changes in youth with severe obesity as currently designed. Exploring additional treatments that could be combined with the RPMS, such as virtual BFI groups, is an important direction for future modification and evaluation of RPMS for treating pediatric obesity. Third, this pilot open trial did not include a control group, which limits our ability to draw conclusions regarding the initial effectiveness of the RPMS. Future studies examining RPMSs in pediatric obesity clinical treatment settings should include control groups so that changes in weight and lifestyle behavior (e.g., dietary intake, physical activity) outcomes can be compared. Fourth, the generalizability of the RPMS is restricted. Specifically, the RPMS evaluated was designed to treat patients seen in the specific pediatric obesity clinic where the study was conducted. Specifically, participants were youth with severe obesity living in Mississippi who primarily identified as non-Hispanic Black/African American and who were English-speaking. The specific RPMS developed and evaluated may not be applicable to patients being treated in other pediatric weight management clinics located in other areas of the US or in clinics who serve different patients based on race/ethnicity, socioeconomic status, language status, and geographic locations. Future research utilizing larger samples would benefit from examining potential differential RPMS treatment outcomes based on race/ethnicity, sex, and other sociodemographic factors to further examine how the RPMS can be modified to further address health disparities. In addition, translating the information presented in the RPMS into other languages would increase accessibility for non-English-speaking families and increase generalizability and access for under-represented groups subject to health disparities. 

## 6. Conclusions

In summary, this pilot open trial examined usability and satisfaction of an RPMS designed for youth with severe obesity who were racially diverse and from an area of the US that experiences significant health disparities. Participating families expressed satisfaction with the RPMS, but engagement was limited, and the initial effectiveness results revealed limited improvements in outcomes post-treatment. Modification and further evaluation of an RPMS that increases use and effectiveness for treating pediatric obesity are warranted. In the future, a telehealth treatment that is designed for underserved and racially diverse youth and increases connection to health care providers and health information may be a cost-effective and practical weight management treatment for youth with severe obesity, resulting in better long-term health outcomes and reduced health care costs. 

## Figures and Tables

**Table 1:** Youth and Caregiver Demographic Characteristics.

*Youth Characteristics (N = 47)*	*Mean (SD) or n (%)*
Age in years	13.55 (2.21)
Sex (% Female)	27 (57.4%)
BMI	41.57 (11.41)
BMI percentile	99.32 (0.80)
BMI z-score	2.59 (0.41)
Class of Obesity	*n (%)*
Class 1 Obesity (BMI 30 to <35)	12 (25.5%)
Class 2 Obesity (BMI 35 to <40)	12 (25.5%)
Class 3 Obesity (BMI > 40)	23 (48.9%)
Race/Ethnicity	*n (%)*
Non-Hispanic White/Caucasian	10 (27.0%)
Non-Hispanic Black/African American	33 (70.2%)
Latino/Hispanic	3 (6.4%)
Biracial	1 (2.1%)
*Caregiver Characteristics (N = 45)*	*Mean (SD)*
Age in years	42.25 (9.70)
BMI (*n* = 35)	37.66 (9.38)
Relationship to Youth	*n (%)*
Mother	39 (86.7%)
Father	1 (2.24.1%)
Grandmother	4 (8.9%)
Other	1 (2.2%)
Marital Status	*n (%)*
Married	16 (35.6%)
Single/Never Married	17 (37.8%)
Divorced/Separated	3 (6.7%)
Cohabitating	5 (11.5%)
Widowed	4 (8.9%)
Annual Family Income	*n (%)*
Below $9999	6 (13.3%)
$10,000–$29,999	13 (28.9%)
$30,000–$49,999	11 (24.4%)
$50,000–$69,999	6 (13.3%)
$70,000–$89,999	2 (4.4%)
Above $90,000	7 (15.6%)

Note: BMI = Body Mass Index.

**Table 2:** RPMS Use and Satisfaction Scores.

*RPMS Use Information (N = 38)*	*Mean (SD)*	*Median*	*Range (Min–Max)*
Partially Completed RPMS Sessions (*n* = 38)	2.76 (2.57)	2.00	0–9
Fully Completed RPMS Sessions (*n* = 38)	24.53 (22.09)	20.50	0–88
Total RPMS Sessions Completed ^a^ (*n* = 38)	27.29 (22.42)	24.50	0–88
*Youth RPMS Satisfaction (N = 29)* ^b^	*Mean (SD)*	*Median*	*Range (Min–Max)*
Total Youth Satisfaction ^c^ (*n* = 26)	32.88 (5.37)	33.50	16–40
1. I liked using the RPMS. (*n* = 29)	3.21 (0.94)	3.00	1–4
2. The RPMS helped me improve my healthy habits. (*n* = 29)	3.21 (0.86)	3.00	1–4
3. It was easy to answer the questions asked by the RPMS. (*n* = 29)	3.62 (0.73)	4.00	1–4
4. I felt supported when my parent or I talked with the nurse. (*n* = 28)	3.54 (0.58)	4.00	2–4
5. The information presented was interesting. (*n* = 28)	3.50 (0.84)	4.00	1–4
6. After using the system I got along better with my parent. (*n* = 29)	3.00 (1.04)	3.00	1–4
7. Using the RPMS system was time consuming. ^d^ (*n* = 29)	2.69 (1.14)	3.00	1–4
8. I liked the graphics used in the system. (*n* = 29)	3.07 (0.96)	3.00	1–4
9. Overall, the RPMS was easy to use. (*n* = 27)	3.56 (0.75)	4.00	1–4
10. Overall, using the RPMS was positive. (*n* = 29)	3.45 (0.87)	4.00	1–4
*Caregiver RPMS Satisfaction (N = 27)* ^b^	*Mean (SD)*	*Median*	Range *(Min–Max)*
Total Caregiver Satisfaction ^e^ (*n* = 23)	42.26 (4.39)	42.00	32–48
1. I enjoyed viewing the educational materials in the RPMS. (*n* = 27)	3.41 (0.64)	3.00	2–4
2. Using the RPMS helped me feel responsible for my family’s lifestyle behaviors. (*n* = 27)	3.30 (0.72)	3.00	1–4
3. The RPMS made me feel that I am not alone in my family’s struggle to develop healthier lifestyles and manage weight. (*n* = 27)	3.48 (0.64)	4.00	2–4
4. I felt comfortable answering the questions asked by the RPMS. (*n* = 27)	3.63 (0.56)	4.00	2–4
5. Using the RPMS helped me learn how to overcome barriers to a healthy lifestyle. (*n* = 27)	3.30 (0.61)	3.00	2–4
6. The information presented was applicable to my child and family. (*n* = 27)	3.48 (0.58)	4.00	2–4
7. The information collected by the system (physical activity, weight) was helpful to me and my child. (*n* = 27)	3.59 (0.64)	4.00	2–4
8. Overall, I feel using the RPMS has been a positive experience. (*n* = 27)	3.74 (0.45)	4.00	3–4
9. The telehealth nurse understood the unique challenges and needs of my family in making healthy lifestyle changes. (*n* = 23)	3.57 (0.59)	4.00	2–4
10. I felt that the telehealth nurse appreciated and respected the small changes my family made while using the program. (*n* = 23)	3.57 (0.59)	4.00	2–4
11. I learned helpful ways to improve my child’s behavior while using the RPMS. (*n* = 27)	3.26 (0.71)	3.00	2–4
12. The RPMS was easy to use. (*n* = 27)	3.63 (0.56)	4.00	2–4

Notes: RPMS = Remote Patient Monitoring System. ^a^ Total RPMS Sessions Completed includes the partially complete and fully complete sessions. Total possible RPMS Sessions is 90. ^b^ Items were answered on a 4-point Likert scale. ^c^ The total possible Youth RPMS Treatment Satisfaction score was 40, with higher scores indicating more satisfaction. ^d^ Question 7 on the Youth Treatment Satisfaction questionnaire was reverse coded. ^e^ The total possible Caregiver RPMS Treatment Satisfaction score was 48, with higher scores indicating more satisfaction.

**Table 3:** Pre-Treatment and Post-Treatment RPMS Comparisons and Statistical Results.

	*Pre-RPMS* *M (SD)*	*Post-RPMS* *M (SD)*	*Wilcoxon Signed Ranks Test Value*	*p-Value*
*Youth Assessments and Questionnaires*				
BMI (*n* = 22)	39.52 (6.63)	40.37 (6.33)	−0.817	0.414
BMI Percentile (*n* = 20)	99.13 (1.08)	99.06 (0.68)	−1.717	0.086
BMI z-score (*n* = 20)	2.48 (0.44)	2.54 (0.32)	−0.104	0.918
Heart Rate (*n* = 17)	81.24 (10.17)	87.81 (14.89)	−1.753	0.080
Systolic Blood Pressure (*n* = 17)	118.47 (11.51)	125.76 (12.83)	−1.847	0.065
Diastolic Blood Pressure (*n* = 17)	68.62 (7.37)	73.18 (8.10)	−1.564	0.118
ASA24 dietary intake kilocalories/day (*n* = 14)	1427.75 (529.50)	1289.35 (576.01)	−0.785	0.433
Actigraph physical activity kilocalories/day (*n* = 15)	384.81 (236.18)	234.17 (215.54)	2.442	0.015 *
CDSES Total Score (*n* = 25)	37.12 (6.04)	39.24 (5.09)	−1.934	0.053
SEPAS Total Score (*n* = 28)	10.32 (2.16)	10.79 (2.61)	−0.734	0.463
PedsQL Total Score (*n* = 27)	68.60 (15.64)	74.40 (15.97)	−2.114	0.035 *
*Caregiver Assessments and Questionnaires*				
BMI (*n* = 12)	36.78 (8.96)	37.03 (9.87)	−0.078	0.937
HFI Obesogenic Home Food Availability (*n* = 20)	22.80 (7.66)	28.25 (11.89)	−1.963	0.050 *
PedsQL Total Score (*n* = 19)	72.31 (13.09)	79.18 (13.12)	−1.853	0.064

Notes: RPMS = Remote Patient Monitoring System; BMI = Body Mass Index; ASA24 = Automated Self-administered 24-h Recall (ASA24); CDSES = Child Dietary Self-Efficacy Scale; SEPAS = Self-Efficacy for Physical Activity Scale; PedsQL = Pediatric Quality of Life Inventory; HFI = Home Food Inventory. * *p* < 0.05.

## Data Availability

The data presented in this study are available on reasonable request from the corresponding author.
